# Anchoring of a Single Molecular Rotor and Its Array on Metal Surfaces using Molecular Design and Self-Assembly

**DOI:** 10.3390/ijms11020656

**Published:** 2010-02-09

**Authors:** Li Gao, Shi-Xuan Du, Hong-Jun Gao

**Affiliations:** Institute of Physics, Chinese Academy of Sciences, Beijing 100190, China

**Keywords:** molecular rotor, molecular self-assembly, scanning tunneling microscopy

## Abstract

Functionalizing of single molecules on surfaces has manifested great potential for bottom-up construction of complex devices on a molecular scale. We discuss the growth mechanism for the initial layers of polycyclic aromatic hydrocarbons on metal surfaces and we review our recent progress on molecular machines, and present a molecular rotor with a fixed off-center axis formed by chemical bonding. These results represent important advances in molecular-based nanotechnology.

## 1. Introduction

Molecules are important building blocks for bottom-up fabrication of functional nanostructures in nanotechnology [1–3]. Integrating device functions onto single molecules will be an ideal solution for the device miniaturization pushed by the Moore’s Law. Most molecules can self-assemble into ordered nanostructures on surfaces, which can be precisely controlled through modifying the properties of molecules or surfaces [4–15]. Molecular self-assembly can produce high-quality functional organic thin films and well-ordered organic/metal interfaces, and in the meanwhile, is a feasible scheme for fabricating complex nanoscale circuits. The most important advantage of molecules lies in the fact that the well-developed molecular synthesis techniques can produce various molecular structures, which offers a high controllability over single molecular properties and molecular nanostructures. In particular, molecular self-assembly and single molecular machines are two of the most crucial issues in molecule-based nanotechnology. Polycyclic aromatic hydrocarbons (PAHs) and metal phthalocyanines (Pcs) are among the most promising candidates for molecular-based nanotechnology.

In this review, we discuss the growth mechanism for the initial layers of PAHs on metal surfaces, as well as the functional units realized on single molecules. Perylene and tetra-*tert*-butyl zinc phthalocyanine [(*t*-Bu)_4_-ZnPc] molecules are used to discuss these two issues, respectively. Our discussions are mainly based on the results obtained by a scanning tunneling microscope (STM) [16], which is a unique tool in surface science, and excels in high-resolution topographic imaging, scanning tunneling spectroscopy, spin-polarized measurements, atomic or molecular manipulation, nanolithography, single molecule optical spectroscopy, chemical modification, and so on.

## 2. Growth Mechanism for Polycyclic Aromatic Hydrocarbons Initial Layers on Metal Surfaces

The growth process of the first PAHs monolayer on metal surfaces is inherently a non-equilibrium phenomenon governed by the competition between kinetics and thermodynamics [1]. The diffusion of a molecule on a flat terrace is the most important kinetic process in the monolayer growth. The surface diffusion coefficient *D* is related to the site to site hopping rate of a molecule *k_s_* by *D* = *a*^2^*k_s_*, where *a* is the effective hopping distance between sites and *k_s_* is proportional to exp(−*V_s_*/*k_B_T*), where *V_s_* is the potential-energy barrier from site to site, *T* is the substrate temperature, and *k_B_* is the Boltzmann constant. If molecules are deposited on the metal surface at a constant deposition rate *F*, then the ratio *D*/*F* determines the average distance that a molecule has to travel to arrive at its final adsorption site before the coverage is increased up to one monolayer, because PAHs do not aggregate into molecular islands in the submonolayer coverage range. Therefore, the ratio of deposition to diffusion rate *D*/*F* is the key parameter of characterizing growth kinetics. In the case of large values of *D*/*F*, growth occurs close to equilibrium conditions; that is, the molecules have enough time to explore the potential energy surface so that the system reaches a minimum energy configuration. On the contrary, in the case of small values of *D*/*F*, the growth is essentially determined by kinetics; individual processes, especially those leading to metastable structures, are increasingly important.

**Figure 1.**
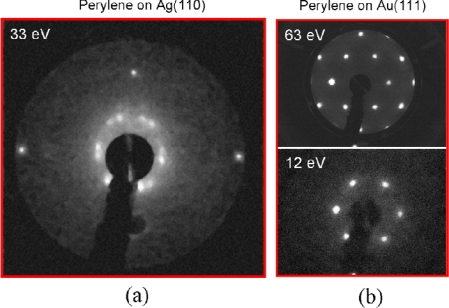
(a) LEED pattern of a highly ordered perylene monolayer on Ag(110). (b) LEED pattern of Au(111) substrate at 63 eV, and that of perylene monolayer on Au(111) at 12 eV.

Molecules were deposited onto metal surfaces from the gas phase in ultra-high vacuum (UHV), which is referred to as organic molecular beam deposition (OMBD) or organic molecular beam epitaxy (OMBE), and is an ideal means to achieve a high-level control over the molecular deposition with extremely high chemical purity and structural precision. In our experiments, molecules were deposited with a MBE-LEED apparatus, which integrates molecular beam epitaxy with low energy electron diffraction and allows *in situ* recording of diffraction patterns in real time during molecular deposition. For the deposition of PAHs on metals, the LEED pattern appears like a halo at dilute coverage, changes into a diffuse ring with the coverage increasing, then decays into diffuse spots with the coverage close to one monolayer, and finally shows sharp diffraction spots at monolayer coverage. Figure 1(a), (b) are the LEED patterns of perylene monolayer on Ag(110) and on Au(111), respectively [6–8].

Thermal evaporation was performed with the substrate being kept at around room temperature. However, STM observations were conducted after the samples were cooled down to low temperatures, 78 K or 7 K, in order to get molecules frozen and thus better imaged. In the slow cool down process, molecules can find their favorable adsorption sites on the surface. In the following, we summarize our low-temperature STM observations of the adsorption of perylene on metal surfaces.

At dilute coverage, molecules can only be observed at step edges [7]. When a small number of perylene molecules were deposited onto the silver surface at 320 K, most of the molecules adsorbed on step edges, while only a few molecules were observed on terraces, which indicates that the sites at step edges on Ag(110) are more active, compared with the sites on terraces, for the adsorption of perylene at 78 K [7]. All molecules along step edges lie below step risers in the form of isolated molecules without uniform intermolecular distance. The configuration of the molecules adsorbed on step edges is different from the “flat-flying” configuration on terraces due to the existence of the microfacets at steps edges. Surface steps, in many cases, are the preferential adsorption sites for molecules. The diffuse halo-like LEED pattern appears and expands from the center when the coverage further increases [7]. The expansion of the halo is the result of increasing the coverage and thus decreasing the average intermolecular distance. After the step edges are filled completely, molecules start to adsorb randomly on the terraces without ordered arrangement. Quasi-ordered molecular arrangements were observed when the coverage was increased close to one monolayer. Highly ordered arrangements were obtained at monolayer coverage. These STM results are in agreement with the transition of the LEED pattern recorded during the molecular deposition. The ordered superstructure is mainly induced by the intermolecular interaction at monolayer coverage. Such mode of monolayer growth is typical for PAHs on metal surfaces [6–8].

**Figure 2.**
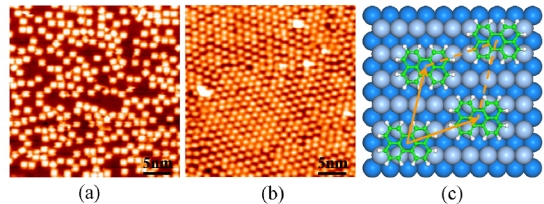
**(a)** STM image (35 nm × 35 nm, *U* = −1.8 V, *I* = 0.04 nA) showing the scenario after one submonolayer of perylene, with a molecular coverage of 3.1 × 10^13^ molecules per cm^2^, was cooled down to 7 K. **(b)** High-resolution STM image (30 nm × 30 nm, *U* = −0.9 V, *I* = 0.21 nA) of perylene monolayer on Ag(110). One domain boundary is shown in this image. The image was taken at 78 K. **(c)** The model of the favorable superstructure obtained with energy optimization.

Figure 2(a) is a typical STM image showing the scenario after the sample was cooled down to 7 K. The submonolayer was fabricated by depositing about 3.1 × 10^13^ molecules per cm^2^ on the substrate at 320 K. It can be seen that all the molecules distribute on the silver surface homogeneously and disorderly at this coverage. Extensive STM images show that the molecules on step edges do not act as capturing nucleus for further growth from steps during the cooling process because no condensed molecular islands are observed near steps. On terraces, molecules do not aggregate into condensed molecular islands either. This growth mode is ascribed to the weakness of intermolecular interaction, and typical for weakly interacting systems.

Figure 2(b) shows the STM image of highly ordered perylene monolayer that was fabricated by evaporating about 5.0 × 10^13^ molecules per cm^2^ onto the Ag(110) surface at 320 K. The structure of the monolayer was determined by analyzing LEED pattern at room temperature and high-resolution STM images at 78 K. To capture the diffraction spots of the perylene superstructure and those of the silver substrate in one picture, the electron energy was increased up to 33 eV. Figure 1(a) shows the captured LEED pattern, including twelve diffraction spots of perylene superstructures and four of silver substrate. The molecular superstructure has two domain orientations mirrored at the crystal axis of the Ag(110) substrate, which is induced by the two-fold symmetry of the substrate. Approximately, a commensurate superstructure is determined by the LEED pattern [7]. STM results at 78 K indicate the same superstructure. In many cases, the difference in the thermal expansion coefficient of the metal substrate and the organic adlayers leads to structural transitions with increasing temperature. However, such a transition was not observed in our experiments.

Our STM experiments show that the size of single domain is far larger than 115 nm, which is close to the upper limit of the scanning range for molecular resolution; that is, the density of domain boundary is rather low. Figure 2(b) shows the domain boundaries. Even near these boundaries, the highly ordered arrangement is maintained, and only several molecules are mismatched.

Figure 2(c) shows the favorable adsorption configuration for perylene on Ag(110) surface and the monolayer superstructure can be described as:

$$\text{perylene}=\left(\begin{array}{ll} 4 & 1\\ 1 & 3 \end{array}\right)\text{Ag}(110)$$

**Figure 3.**
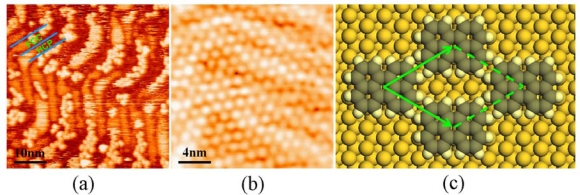
**(a)** STM image (50 nm × 50 nm, *U* = −1.3 V, *I* = 0.05 nA) of 0.3 ML perylene molecules on the reconstructed Au(111) surface. **(b)** STM image (20 nm × 20 nm, *U* = −0.7 V, *I* = 0.03 nA) of perylene monolayer. **(c)** Supposed structural model for perylene monolayer on Au(111) surface. Bridge site is selected according to our DFT calculations.

Figure 3(a) shows the STM image of 0.3 ML perylene molecules on Au(111). Some bright molecular aggregates were observed in our STM measurements at 78 K. The molecular aggregates can hardly diffuse over the surface but vibrate locally, thus they can be imaged as bright aggregates. Ordered molecular arrangements form when the coverage increases up to one monolayer, as shown in Figure 3(b). Combining LEED results and STM measurements, we determined the monolayer lattice as a (4 × 4) structure [8]. In the STM images, individual perylene molecules extend ~1 nm in the [11 2] direction and ~0.6 nm in the [1 10] direction [8]. This indicates that the molecular long axis is along the [11 2] direction. Figure 3(c) depicts the supposed model for a perylene monolayer on a Au(111) surface. According to our theoretical calculations, the bridge site is the most stable adsorption site [8]. The monolayer superstructure of perylene on Au(111) can be described as:

$$\text{perylene}=\left(\begin{array}{ll} 4 & 0\\ 0 & 4 \end{array}\right)\text{Ag}(111)$$

Symmetry determines the domain number for the molecular superstructure on substrate. Generally, molecules and substrates are chosen to meet the requirements for epitaxial growth with single domain orientation. For perylene on Ag(110), the two-fold symmetry of the Ag(110) surface leads to two domain orientations mirrored at a crystal plane of the Ag(110) substrate. In contrast, for perylene on Au(111), single domain orientation was observed because both the Au(111) surface and the molecular superstructure show a six-fold symmetry. Single domain orientation is desirable for organic thin films applied in devices.

Molecular self-assembly is driven by the surface structure of the substrate and the balance between intermolecular and molecule-substrate interactions. In some cases, the surface structure can serve as a template for the formation of one-dimensional or two-dimensional molecular nanostructures [17]. In other cases, the molecular self-assembly on flat and unstructured surfaces can be realized by strong molecule-substrate interaction or intermolecular interaction [18]. For some organic/metal systems, such as perylene/Ag(110), the molecule-substrate interaction, the intermolecular interaction and the template-influence of the substrate are all quite weak, in which case the fabrication of highly ordered molecular nanostructures is quite sensitive to growth conditions due to the lack of an internal dominating driving force for self-assembly. In our experiments, when the deposition rate increased from ~4 × 10^−3^ ML/min, the rate for the uniform monolayer, to ~1 × 10^−2^ ML/min, many metastable structures appeared. In addition, Seidel *et al.* reported some non-commensurate superstructures for perylene on Ag(110) obtained under slightly different growth conditions [19]. The formation of non-commensurate superstructures is another evidence that the interaction between perylene molecules and Ag(110) substrate is weak.

**Figure 4.**
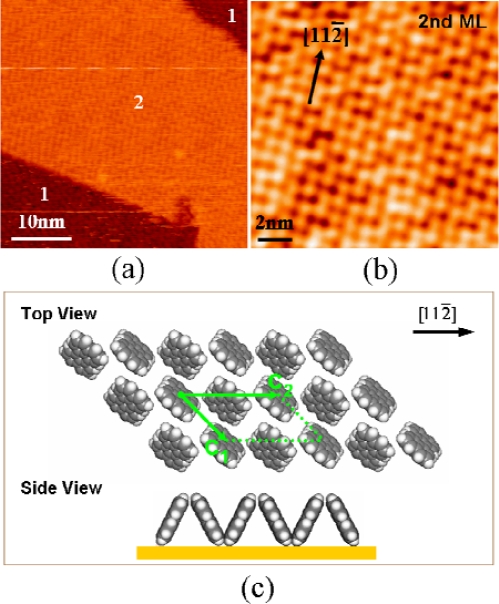
**(a)** STM image (40 nm × 40 nm, *U* = −0.4 V, *I* = 0.04 nA) showing the molecular arrangements of both the first layer “1” and the second layer “2” of perylene on Au(111). **(b)** STM image (15 nm × 15 nm, *U* = −0.4 V, *I* = 0.04 nA) of the second layer of perylene on Au(111). **(c)** The proposed structural model of the second layer. Side view is along **c**_**1**_ direction.

The growth of the second layer is completely different from that of the first layer. When additional molecules are evaporated onto the first layer, two-dimensional molecular islands form with an ordered arrangement, as shown in Figure 4(a). The sticking coefficient for the molecules impinging on the first monolayer is much lower than that for the molecules impinging on the gold surface. Only a small number of molecular islands were observed after further depositing molecules onto the first monolayer using the same incident flux for 100 minutes, the typical growth time for the first monolayer. The apparent height of the molecular islands is 4–5 Å, varying with the tunneling conditions in STM scanning. In contrast, the apparent height of “lying-flat” perylene molecules is 1–1.5 Å. Figure 4(b) represents a typical STM image showing the molecular arrangement of the molecular island. Molecules assemble in a dimer-like arrangement in the [11 2] direction. The structure of the second layer can be determined. Molecules form an oblique two-dimensional Bravais lattice with two molecules at each Bravais lattice point. We determine that the molecules in the second layer adopt a tilted configuration, instead of a flat-lying configuration, according to the measured height of the islands and the molecular density derived from the STM measurements. Figure 4(c) illustrates the supposed structural model of the second layer.

Some previous studies show that perylene molecules assemble with the π-plane oriented almost or completely parallel to the substrate in the multilayer region [20,21]. Other studies show that a planar or near-planar orientation of perylene molecules is limited to the first monolayer, and the transition to the bulk structure occurs with increasing film thickness [22,23].

The difference in growth mode between the first two layers is ascribed to their different environment with respect to the competition between molecule-molecule and molecule-substrate interactions. The dominant force for the growth of the first layer is the molecule-substrate interaction. The growth of the second layer is dominated by molecule-molecule interaction, which results in the growth of ordered molecular islands. The existence of the first layer leads to a remarkable decrease of the interaction between the substrate and the molecules of the second layer. We have observed similar growth phenomenon for iron phthalocyanine molecules on Au(111) surface [9].

The growth mode for perylene on Au(111) was also observed for many other PAHs [22]. Generally the planar PAHs molecules form a monolayer on metal surfaces with the molecular plane parallel to the substrate [24]. In most cases, including perylene/Au(111), however, the bulk structure of PAHs does not include a low-index and closely packed plane whose molecular arrangements are the same as the molecular monolayer on metal surface. From the second layer onward, perylene films adopt a different packing arrangement from that of the first monolayer, and get close to the bulk structure. For perylene/Au(111), the molecular island is an intermediate structure, with molecules tilting from the surface and forming a dimeric arrangement. The surface structure of an epitaxial perylene multilayer crystal is the herringbone packing of dimeric perylene molecules in the *ab-*plane of the monoclinic α-perylene single crystal [22]. Although the structure of the molecular island in the second layer is different from the final multilayer structure, the former has two features of the latter, *i.e.*, dimeric arrangement and a tilting molecular orientation.

Metallic surfaces are thermodynamically unstable in a cleaved-bulk configuration, and some of them reconstruct into an atomic arrangement different from the bulk one. The bare Ag(110) surface has the same atomic arrangement as the bulk crystal. In contrast, the bare Au(111) surface shows a herringbone reconstruction pattern exhibiting an ordered array of domain boundaries between surface regions with different atomic stacking, face-centered-cubic (fcc) and hexagonal-close-packed (hcp) stacking. Such surface reconstruction structures have great effect on the molecular adsorption, especially for submonolayer coverage. The adsorption locations of all aggregates are very selectively influenced by the surface reconstruction of the Au(111) substrate. The fcc region is more favorable than the hcp region for perylene, as shown in Figure 2(a).

## 3. Single Molecular Functional Units on Metal Surfaces

Fabricating high-quality organic thin films on metal surfaces, as discussed above, has started to be applied in organic electronics with advantages of low cost and high flexibility, and has more promising applications in nanotechnology [25–34]. However, realizing complex functions at a molecular scale is the ideal solution for device miniaturization. Recently, the door towards molecular electronics is being pushed open a little further, thanks to considerable efforts on STM studies of single molecules on surfaces. With its capability of high-spatial-resolution and high-energy-resolution measurements, STM has helped reveal many interesting physics within single molecules, including electron transport [35–37], spin-flip excitations [38,39], vibrational excitations [40] and mechanical motions [41–43].

The motion of single atoms or molecules plays an important role in nanoscale engineering at the single atomic or molecular scale [44,45]. The controllability of molecular motion is critical for molecular motors [46], which may convert external energy into orchestrated motion at the molecular level [47,48]. For molecular rotors [49] a high level of control over the rotation axis and, equivalently, self-assembly on a very large scale, are the key ingredients for their integration into complex molecular machines. In addition, the studies mainly focused on single molecules, while it is desirable, for eventual applications, that individual molecular rotors self-assemble into large scale ordered arrays while keeping their original functions. Our recent studies [43] show, using STM [50–52], that single tetra-*tert-*butyl zinc phthalocyanine [(*t*-Bu)_4_-ZnPc] molecules on the reconstructed Au(111) surface possess a well-defined rotation axis fixed on the surface, and also, that these single-molecule rotors form large scale ordered arrays due to the reconstruction of the gold surface. Gold adatoms at the surface function as the stable contact of the molecule to the surface. An off-center rotation axis is formed, by a chemical bonding between a nitrogen atom of the molecule and a gold adatom on the surface, which gives them a well-defined contact while the molecules can have rotation-favorable configurations.

**Figure 5.**
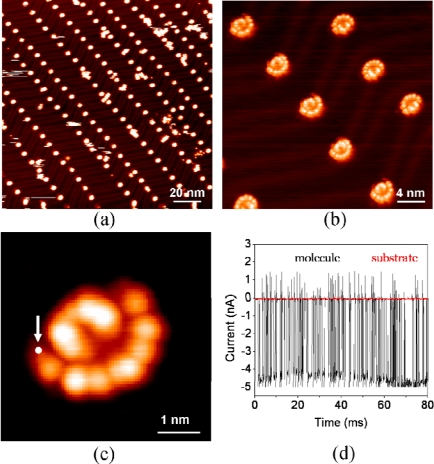
**(a)** STM image of large scale ordered array of single (*t*-Bu)_4_-ZnPc molecular rotors on the reconstructed Au(111) surface. **(b)** High-resolution STM image of single molecular rotors showing a “folding-fan” feature. The molecular rotors at two different elbow sites show different features due to the modulation by corrugation ridges. (*U* = −1.3 V, *I* = 0.07 nA). Images were taken at 78 K. **(c)** A close-up high-resolution STM image of single molecular rotor showing a “folding-fan” feature. (*U* = −2 V, *I* = 0.05 nA). (d) Tunneling current versus time measured on the molecular rotor. The arrow and spot in (c) indicate the position where the *I-t* spectroscopy was measured.

Figure 5(a) shows an STM image of a large scale array of (*t*-Bu)_4_-ZnPc molecules on Au(111). The molecules adsorb predominantly at the elbow positions of the surface reconstruction, typical for Au(111) surfaces. The $22\times \sqrt{3}$ surface reconstruction in this case acts as an atomically precise template for the selective adsorption of (*t*-Bu)_4_-ZnPc molecules on the Au(111) surface. High-resolution images [see Figure 5(b),(c)] reveal a feature of the adsorbed molecules that is reminiscent of a folding fan. The “folding-fan” feature cannot be observed at 5 K. We propose that the “folding-fan” feature is actually the low-frequency image of a high-frequency molecular rotation on Au(111), driven by thermal energy.

To verify that the “folding-fan” is caused by molecular instability with respect to the substrate surface, we monitored the tunneling current versus time locating the STM tip at a fixed point on the “folding-fan” [see Figure 5(c)], applying a constant bias voltage of −1.8 V to the sample, and recording the tunneling current as a function of time. Figure 5(d) shows the recorded tunneling current within an interval of 80 ms. The amplitude of the tunneling current oscillates with a high frequency between 0 and 5 nA. The oscillation indicates that the “folding-fan” feature is really due to rapid molecular motion, at the same time excluding the possibility of impurities or other artifacts.

Only one (*t*-Bu)_4_-ZnPc molecule is involved for each “folding-fan” feature. In our experiments stationary dimers, trimers, tetramers, and larger clusters of (*t*-Bu)_4_-ZnPc can be observed at 78 K. In contrast, a stationary single (*t*-Bu)_4_-ZnPc molecule, whose STM image should be composed of four lobes, cannot be observed at 78 K. This indicates that single molecules are not stationary but unstable on the surface at this temperature. Besides that, in our experiments we observed the transition from the stationary state to the unstable state for single (*t*-Bu)_4_-ZnPc molecule, and found that single molecule remained stationary when it was attached to the clusters, but it became unstable showing the “folding-fan” feature as soon as it was isolated.

We propose that the “folding-fan” feature is induced by the rotation of single (*t*-Bu)_4_-ZnPc molecule. The existence of a rotation center is the prerequisite for rotation, rather than lateral diffusion along the surface at elevated temperatures. Since the center of the STM image of the molecular rotors is dark, the rotation center cannot be at the position of the *tert*-butyl groups that appear as bright protrusions in STM measurements. Our STM observations combined with the first principle calculations reveal that the most likely rotation center is the gold adatom on the surface. Generally, the array of the single molecular rotors in Figure 5 is very stable, but occasionally one molecule was removed during the scans leaving a small bright spot at the center position of the previous molecular rotor [43]. This phenomenon has been observed several times. The bright spot, observed after the removal of a single molecule, is most likely a gold adatom. Gold adatoms on the reconstructed gold surface are stable at 78 K [53], and capable of enhancing the interaction between the adsorbed molecule and the surface, forming a potential well that prevents lateral diffusion of the molecule along the surface. The gold adatoms are formed in the sample cleaning process and prefer to adsorb at the elbow sites at 78 K. In our experiments at 78 K, we did not find isolated single molecules that do not have the gold adatoms. This implies that the single molecules diffuse over the surface until they attach to a gold adatom or a molecular cluster.

First-principles calculations were carried out in order to find out the role of the gold adatom in the molecular adsorption. Our calculations were based on density functional theory (DFT), a Perdew-Burke-Ernzerhof (PBE) generalized gradient approximation (GGA) for exchange-correlation energy [54], projector augmented waves (PAW) [55,56], and a plane wave basis set as implemented in the Vienna *ab-initio* simulation package (VASP) [57,58]. A c(5 × 8) supercell was employed to model the isolated molecule on the gold surface. Due to numerical limitations and the size of the system, the surface Brillouin zone of the three-layer gold film was sampled with the Γ-point only. The cut-off energy for the plane waves was 400 eV. In structural relaxations, all atoms except for the bottom two gold layers were fully relaxed until the net force on every atom was smaller than 0.02 eV/Å. According to our calculation, the gold adatom is at the hollow site.

**Figure 6.**
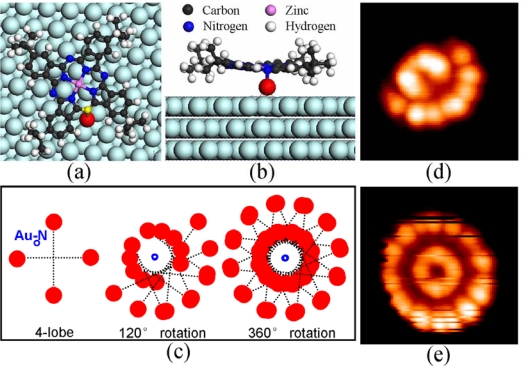
**(a)** Top view and **(b)** side view of the optimized configuration of a (*t*-Bu)_4_-ZnPc molecule adsorbed on the Au(111) surface via a gold adatom. The molecular formula of (*t*-Bu)_4_-ZnPc is C_48_H_48_N_8_Zn. **(c)** Schematic STM images of molecular rotors with rotation angles of 120° and 360°. The red solid circles represent the bright lobes for stationary single molecules. The blue empty circle represents the rotation center. **(d)** STM image of molecular rotors located at the elbow position. **(e)** STM image of molecular rotors located in hcp region.

Figure 6(a,b) is the top and side view of the optimized configuration for a single (*t*-Bu)_4_-ZnPc molecule adsorbed on a gold adatom. Here, our calculations show that the distance between the zinc atom and its nearest neighboring gold atom is 4.60 Å, the distance between the bottom nitrogen [colored in yellow in Figure 6(a)] and the gold atom is 2.25 Å; the adsorption energy of this configuration in this case is 804 meV. In contrast, the calculation results [43] for a single (*t*-Bu)_4_-ZnPc molecule adsorbed directly on Au(111) show that the distance between the zinc atom and its nearest-neighbor gold atom is 4.35 Å, the distance between the bottom nitrogen atom and its nearest-neighbor gold atom 4.40 Å; the adsorption energy of this configuration is 219 meV. Obviously, the gold adatom enhances the molecular bonding significantly, which is most likely due to the surface dipole originating from smeared out electron charge at the position of the adatom [59]. The strong chemical bond between nitrogen and the gold adatom prevents lateral molecular diffusion along the surface, and in particular offers a fixed off-center axis for the rotation of single (*t*-Bu)_4_-ZnPc molecules at 78 K.

We observed that the molecular rotation depends to a large extent on the surface atomic arrangement. It is well known that the Au(111) surface reconstructs into a herringbone structure dividing the surface into four types of regions with different arrangements of surface atoms [60,61]: face centered cubic (fcc), hexagonal close packed (hcp), corrugation ridges, and elbow sites. For the array of molecular rotors all single molecular rotors are located at the elbow sites, showing a “folding-fan” feature. In contrast, the STM images of the molecular rotors located in the fcc region, in the hcp region, and on the corrugation ridges, show “flower” features [43]. Both the “folding-fan” and “flower” features cannot be observed at 5 K. Previous studies reported that molecular rotation can be controlled by chemical environment, ultraviolet or laser light, temperature, and STM manipulation. Our results reveal a novel scheme for controlling the molecular rotation with atomic precision. Here, the variation of the position of surface atoms leads to a redistribution of potential barriers for molecular rotation.

Figure 6(c) shows our model for the rotation of single (*t*-Bu)_4_-ZnPc molecule. The rotation center is the gold adatom, chemically bonded to a nitrogen atom in the molecule. On flat Au(111) surface, there are twelve stable adsorption configurations that are 30 degrees apart from each other and can be interpreted as intermediate states for the molecular rotation. The differences in adsorption energies between these stable configurations are only tens of meV. The molecule switches between them with high frequency under thermal excitation. Since the *tert*-butyl groups are imaged as the bright lobes in STM measurements, the ensuing STM image is the “flower”. The proposed STM image for 360° rotation [see Figure 6(c)] is in good agreement with the “flower” features observed in our experiments [see Figure 6(e)]. The rotation of single (*t*-Bu)_4_-ZnPc molecule at the elbow sites can also be interpreted based on the model for the rotation in the fcc region. The corrugation ridges form barriers for the molecular rotation at the elbow sites. The molecular rotation is limited within an angle of 120° due to the bending of the corrugation lines, which leads to the “folding-fan” feature. The proposed STM image for 120° rotation [see Figure 6(c)] is in good agreement with the experimental STM image of single molecule at the elbow sites [see Figure 6(d)].

The model is in good agreement with the experimental measurements. The experimentally measured distance between the rotor center and the bright lobes on the outer torus is 1.3~1.4 nm, in reasonable agreement with the distance between the nitrogen atom and the *tert*-butyl groups (1.10 ± 0.05 nm), considering that the rotation center is the gold adatom which is not exactly under the nitrogen atom [see Figure 6(a)]. Since hcp and fcc regions of the surface have the same symmetry with respect to the rotation axis, the molecular rotors in the two regions have identical STM images. The molecular rotation on the corrugation ridges can also be understood within this model, because in this case the corrugation ridges modulate the molecular rotors. The corrugation ridges act as the barriers for the molecular rotation at the elbow sites, leading to the “folding-fan” feature. There are two types of elbow sites arranging alternately along the corrugation ridges. The molecular rotation is limited in the fcc region at one type of elbow site, but in the hcp region at the other type of elbow site [see Figure 5(b)].

## 4. Conclusions

Exploring the self-assembly mechanism of PAHs on metal surfaces at single molecular level by STM provides versatile and valuable information for the development of molecular devices. It is of fundamentally important to obtain a full understanding of the self-assembly of nano-structures and the interface properties between the nanostructure and the substrate through a pertinent combination of advanced experimental techniques and first principle calculations. It is inspiring that some functionality has been observed for single molecules, like single molecular rotors discussed in this review. We observed single molecular rotation with an off-center rotation axis fixed to surfaces, and achieved self-assembly of molecular rotors into large-scale arrays. Designing the chemical bond between an atom of the molecule and an adatom on the surface we not only achieved a fixed rotation axis at the surface, but also a spin of the molecule around an off-center axis. The gold adatom, which provides the fixed rotation axis, can be used as an atomically well-defined electrode. A fixed rotation axis off-centre is an important step towards the eventual fabrication of molecular motors or generators.
